# Cases of Fibrous Tumor of the Upper Jaw—Epulis

**Published:** 1846-09

**Authors:** 


### Collectanea.

***Cases of Fibrous Tumor of the Upper Jaw—Epulis.***—J. W. Hawkins, in a clinical lecture, delivered at St. George's Hospital, the substance of which appeared in the *Medical Gazette*, relates the following case and its treatment:

I have placed before you, gentlemen, to-day, the preparations from two cases which you saw operated on on Thursday last: one of them is a fibrous tumor of the palate, removed by Mr. Keate; the other is a fibrous tumor of the upper jaw, which I removed myself: the latter is called an epulis, as being like the gum, and partly growing from it, and I propose to make this disease the subject of our first consideration; looking upon it, however, as essentially the same as Mr. Keate's tumor of the palate, and not as a disease, strictly speaking, of the gum alone, as you might understand from its name. I will read you the history of the first case:

1. Mary Tyrrell, ætat. 30, was admitted, under my care, on the 8th of this month, with a tumor, about the size of a large walnut, or rather larger, of an irregular form, growing from the alveolar process of the right side of the upper jaw, and attached, to all appearance, by a broad base, to that process, in a space intervening from just behind the canine tooth, as far back as the last molar. The tumor is vascular, and at times painful; a portion of it projects internally from the attachment of the alveolar process, and lies (as ascertained by the probe) in contact with the mucous membrane of the hard palate, but is not attached to it, whilst a smaller portion, also unattached, turns upwards over the middle of the external surface of the maxillary bone. The surface of the tumor below this portion is vascular and ulcerated, and projects downwards, and lodges, when the mouth is closed, upon the dorsum and side of the tongue. Although the jaws can be closed, the tumor very much impedes mastication and deglutition; it never bleeds, and is painful only at times. Her health seems to be good.

She has been subject to toothache on that side for the last two years, and at the beginning the pain was very severe; when the tooth had partly loosened from its cavity in the socket, a small lump appeared, about the size of a pin's head, on the outer side of the gum, in the situation of the second bicuspid or first molar tooth; she picked it off, but in one or two months it appeared again in the same place, and has been since growing to its present size; she had one bicuspid and a molar tooth removed by a surgeon about three months ago, which were sound and not loose, according to her account, and a decayed stump was also extracted a day or two after her admission. The tumor has much increased in size lately.

It is said by some surgeons that epulis is more common in the lower jaw, but there are before us several specimens of the disease from either jaw, and I have myself seen and operated, I think, on nearly an equal number in the upper and lower jaws; so that I do not know that it is more frequent in one than in the other.

It is said, also, that this tumor is generally seen in children or young persons; I have myself seen it several times in children, but the majority have been adults like our present patient, and like several of those from whom the tumors on the table were removed—thirty, forty, or more years of age; it is probable, therefore, that there is nearly an equal number of young persons and of adults subject to its formation.

If you examine the tumor on the table, you will perceive that it is solid and firm, and distinctly fibrous, both to the naked eye and under the microscope, the fibres in the centre being evidently nearly perpendicular to the surface of the socket from which it derived its origin; this fibrous structure is most dense in the centre, and becomes softer and mixed with more granular structure towards the circumference, where it is covered by the mucous membrane of the gum, which is in some parts warty and very irregular. When you remove such a tumor, you may find the surface of the bone from which it grows scabrous and irregular, or with spiculæ projecting into the base of the tumor, the bone itself being hard and dense, so that you find it useless to endeavor, with a sharp chisel, (as I tried in vain in my patient,) to shave off the outer surface of the bone. In the other tumor, removed by Mr. Keate, some spiculæ of bone projected into it from the bony palate; its fibrous tissue is, however, rather less dense than in the one from the alveolar process, and the mucous membrane of the palate seems quite free from attachment to it, instead of being a part of the tumor, as in the epulis.

Sometimes, as you would expect in a tumor essentially connected with the bone or periosteum, you have spiculæ of bone in the centre of the tumor, separate from the surface of the bone. Here, for example, is one attached only by a pedicle to the gum, in the centre of which is an osseous nucleus. The natural course of change of all fibrous tissues in their transformation, is to have a deposit of bone, as you may see in fibrous tumors of the uterus, or other parts; *a fortiori* you might expect that fibrous growths of the periosteum or bone would have a tendency to some ossific deposit. I regard what is called epulis, then, as a fibrous tumor of the bone, generally of the surface of the bone, which presents much of the appearance of the gum, because it grows into the gum, and all tumors are inclined to resemble in their structure the tissues in which they form; there is a little difference, therefore, between these two fibrous tumors of the gum and palate, and between them and fibrous tumors of the cellular tissue or uterus, or glands, or skin. Look, again, at this preparation of fibrous tumor of the upper jaw, which was removed by Sir Benjamin Brodie, which has affected all the thickness of the bone; the outer part was, to all appearance, a tumor like that of our patient—an epulis; the centre is almost solid bone; and the interior, where it projects into the cavity of the antrum, resembles a mucous polypus, because the mucous membrane is changed on the inside, as the gum is altered on the outside, during the growth of the tumor; the disease having commenced, according to the evidence of pain in the centre of the bone, some months before it projected externally. And another confirmation of this opinion of the origin of epulis from the periosteum and bone is given by what you saw in this very case of Tyrrell's, namely, that the tumor occupies the whole cavity of the alveolus, where there is no gum, and is quite fibrous in the centre; the circumference alone having the appearance of gum, where it approximates to that texture.

You may have remembered, what our notes specify, that the tumor was vascular; it was of a red color like the gum, which is well seen in this drawing, which I had taken from the patient from whom I removed this round tumor with central bone; and you can press out the blood, and see it return again slowly as in the natural structure of the gum. Yet, although well supplied with blood, it often grows very slowly. I had this cast taken from a woman, thirty years of age, who was under Mr. Keate's care in the hospital, with a tumor of the lower jaw, which began as long as eighteen years previously, and it was removed five years afterwards, without having grown larger than a pea; it was removed again eight years after this, by a second operation, when as large as half a walnut; and in five years more had attained no greater a size than you now see; in the third operation a portion of bone was removed by Mr. Keate. In our patient the tumor became larger than in this case, in less than two years, and was rather unusually rapid, but still was little ulcerated or altered. As it proceeds, the tumor grows more rapidly, and is softer and more vascular, and ulcerates, and has irregularities from the pressure of the teeth; and the ulcers are liable occasionally to become sore and painful, and to bleed slightly, and form a fungous growth, something like that of a malignant tumor, and they are sometimes described as being carcinomatous.

I do not believe, however, that there is any reason to think that fibrous tumors, either of this or any tissue, have a carcinomatous nature; or that, even when they ulcerate extensively, and may be fatal from irritation, they have the power of contaminating the adjacent tissues or of affecting the general system by absorption. In this drawing of a very large fibrous tumor of the uterus, or polypus as it is termed, you may see great vessels and cells in its interior, and the irritation of it destroyed the patient, but no part showed evidence of malignant growth.

This fibrous tumor has cells within it of some size, and the outer portion of an epulis, when growing quickly, may have a few cells developed, although in our patient there is only a little greater softness of the outer parts of the tumor. When not condensed, a fibrous tumor may have very large cysts, as in a case where I tapped a woman with what appeared like ovarian tumor, and removed fifteen pints of fluid, which proved, on examination, to be secreted in a cyst of a large fibrous tumor of the uterus, which had several other cysts of smaller size within it. In fibrous tumors of the interior of a bone, also, very great cysts are sometimes developed; but in the epulis even small ones are not common.

Our patient informs us that she suffered much from toothache before the tumor showed itself; and it seems probable that the irritation of unsound teeth may sometimes occasion the growth, as any source of irritation may do in other textures; but it does not appear that this is often the case, for it grows sometimes from the outside of the alveolus, away from the teeth, and pushes them aside, while they continue perfectly sound; and therefore the pain attributed to the teeth, and often leading unnecessarily to their removal with the view of curing the toothache, is really, in many instances, owing to the growth of the tumor, and not the tumor to the irritation of the teeth.

Like almost all solid adventitious structures, an epulis is not much influenced by any remedies you can employ, and as it occasions considerable inconvenience and deformity, as you have witnessed in Tyrrell, and as there is danger of its affecting more and more of the bone to which it is attached, and may in time become unhealthy and ulcerated, its removal should be recommended at an early period. The steps necessary in the operation depend on the size and situation of the tumor, and its connection with the parts around; but do not forget always to direct your attention to the bone itself as the source of the disease.

1. Suppose that the tumor affects one lamina only of the alveolus, either the outer or inner, with the socket perfect, and the teeth sound, and the base a narrow one; it may be sufficient to cut off the tumor down to the gum, and with a sharp chisel to shave off any little spiculæ of bone which are prominent, and watch very carefully for any sign of unhealthiness in the granulations, and if there be any a day or two afterwards, to touch the surface of the bone from which they spring with nitric acid, or some other caustic, of which I will presently speak.

2. If the inner surface of one lamina is affected as well as the outer, so that the tumor pushes the teeth aside, or passes between them, so that the surface of the bone cannot be got at, the tooth or teeth must first be removed in order that the same proceeding may be successfully carried into effect. For example, this drawing and the corresponding preparation are from a patient of mine, a woman forty years of age, in whom a tumor appeared between the left upper canine and bicuspid teeth, attached by a narrow pedicle just within the socket, ulcerated on the surface, and the gum a little diseased on each side of the root; it had begun two years and a half before, and nine months afterwards, when half its present size, it had been cut off, but grew again. I extracted the two teeth, which were separated, and excised the tumor and as much gum as was altered in appearance, and shaved off some of the outer plate with a chisel; once or twice afterwards the surface was touched with nitric acid, and I believe the disease was cured.

3. If both laminæ of the alveolar process are implicated, so that the tumor rises from the bottom of the socket, caustic will not easily reach it so as to effect a permanent cure, though it may do so if freely applied; as, however, tedious exfoliation of perhaps more than is intended will occasionally follow the free use of caustic, a quicker and more certain cure is produced when there is a narrow but deep attachment in the socket, by removing a V shaped piece of the socket, by means of a small key-hole saw placed on each side of the diseased piece of bone, the two incisions meeting at an angle as deep as appears advisable, and a pair of forceps will break off the portion when almost insulated. The remaining bone, when thus sawn, is too well supplied with blood to exfoliate, and it readily granulates so as to fill up in great measure the place of the bone which is lost.

4. If there is a still deeper disease of the bone between its lamellæ, so that several of the teeth are loosened or displaced, and a swelling of the bone itself, or of one or both its coverings, indicates the formation of the tumor within the cancelli, it becomes necessary to remove a considerable piece, as in some of the preparations on the table, in order that no disease may be left behind. In the lower jaw, the whole thickness of the bone sometimes require to be removed by two perpendicular cuts of the saw, which may be made to half divide the bone, and then strong bone-cutters will break through the remainder, more or less being first cut by the saw, according to the age of the patient and the hardness of the bone; and it is quite surprising how little deformity is created by the removal of a portion of the whole substance of the lower jaw. In order that the saw may be conveniently applied, appropriate incisions must be made in the soft parts; if the disease is situated in the chin, it may happen that the thickness of the bone is not too great, if the lower lip is lax and extensile, for the bone to be sawn and cut, and then dissected out, without any incision at all in the lip: and the same may be done in the centre of the upper jaw; but in most cases a semilunar incision is to be made below the chin, and the lip dissected up, for the saw to be applied through the opening, by which means the subsequent cicatrix is concealed by the dress to a certain extent. If the disease is at the side of the lower jaw, a curved incision is to be made along the basis, and the flap raised for the admission of the saw and cutting forceps, without interfering with the circle of the mouth, and the scar is much hidden by the cravat or the cap strings. In every case the teeth should previously be extracted, as the saw will be impeded by any fragment of the dental substance.

5. In fact, however, the simple fibrous tumor, when situated in the lower jaw, need very seldom be removed with the bone down to the base of the jaw, especially in adults, as the new growth extends deeply in the cancelli less frequently in them than in children. A girl, fifteen years of age, was admitted under my care into the hospital, with a spongy and ulcerated tumor of the right side of the lower jaw, extending from the canine to the last molar teeth, about three-quarters of an inch broad, with indentations of the upper teeth upon it, looking as if all the molar teeth were buried in the tumor, but which, by her account have never appeared. The swelling could be felt on each side of the jaw, as if reaching very near the basis, and it began about seven months before, having given her no pain. I removed it in the way that I have mentioned, down to the basis, as it did not appear, on consultation, safe to remove less, and the semilunar incision of the soft parts healed by the first intention, the loss of bone being scarcely perceptible. Yet if you examine the preparation, you will perceive that the fibrous growth did not extend much below the bottom of the alveolus, and I might, in fact, have left the outline of the base of the jaw untouched. Generally the depth of the alveoli, or very little more, is all that is actually diseased, so that the removal of half an inch or three-quarters of an inch in depth from the top of the socket is enough; but, at the same time, so little inconvenience is experienced by the loss of the whole thickness, that you should not hesitate to do so rather than incur any risk of a portion of disease being left behind.

The mode of operating in such cases as these is this: a perpendicular cut is to be made in the jaw on each side of the diseased growth, to the full depth of the part which you intend to remove; then you make a horizontal groove in the bone by means of Hey's saw, in a line at right angles with the former cuts, and then you can cut off the insulated portion of bone by cutting forceps, without the risk of the bone breaking horizontally beyond the proper distance, the depth to which your horizontal cut extends being greater in proportion to the hardness of the bone. The cutting forceps often exhibited in the shops consist, like this, of two equal semicircles, with straight handles, like pincers, when, of course, it is impossible to get the branch within to the right place opposite the outer one; nor does the turning of the handles get them sufficiently out of the way of the teeth of the upper jaw; you can effect this object, however, by the inner branch being twice as long as the outer, by which means its inner extremity curves round the jaw to the proper depth within the mouth. Sometimes a cutting forceps, the branches moving like those of a pair of scissors, but somewhat curved, will answer your purpose, the bone, in either case, breaking up if the groove is made to admit one end of the forceps. A more effective instrument is this which I show you, recently made by Sevigny, where two sharp cutting blades are placed opposite one another, one within and the other without the portion of bone to be removed, and then a handle turns a screw, which quickly forces the outer blade onwards in the groove made horizontally in the bone, and cuts it off with much power, and without any straining or irregular action of the hands, as in the use of the common cutting forceps; it is a very useful instrument in most cases of this kind, and removes the bone easily.

6. For the epulis of the upper jaw, similar proceedings may be adopted, according to the size of the part to be removed, and incisions must be made according to the situation of the tumor; in the centre the lip can be raised without any external incision; at the side the cheek may be opened horizontally, or a flap insulated and turned upwards to expose the necessary extent of the bone. It is very seldom necessary, however, any more than in the lower jaw, to excise more than the depth of the alveolus, or a little more, by a V cut, or by the two perpendicular cuts of the saw, and the separation of the diseased part horizontally by the screw-cutting forceps, which I have shown you. Here is a preparation of the upper jaw, which was removed; yet even here, although the tumor half fills the antrum, you can perceive that the orbitar plate might probably have been safely left.

Now let us apply these remarks to our patient, and see the reason of what I did in her case. It was obvious that the whole breadth and depth of the alveolus was implicated in the base of the tumor, since it overlapped both the palate and the outside of the jaw; but as there was no bulging of the bone above, it was not likely that more than the depth of the alveolus was affected. So much, then, must have been excised, to secure the patient against a return of the disease, but to make the necessary perpendicular and horizontal sections of the bone, an incision must have been made in the cheek, as the disease was too far within the mouth to be otherwise accessible. But then it was possible, by cutting off as much of the basis as could be reached, and subsequently applying caustic, that the origin of the tumor from the surface of the whole depth of the socket might be destroyed; and if only the surface of the bone was affected, that the disease might never return. As, then, this plan was not unlikely, though not certain, to effect a permanent cure, and as when the disease, when it returns after being not perfectly removed, is often very slow in its progress, as I have already shown you, and as the nature of this fibrous growth is innocent, and not likely to be excited into rapid progress, as a carcinomatous growth is sure to be, by being meddled with without complete removal, I gave the patient her choice of what she would have done; and she decided, as I should myself have chosen, I think, to run the chance of the disease returning, and then having the more sure method of operation practised.

In the operation, then, I insinuated the flat surface of the knife between the palatine projection of the tumor of the palate, and afterwards between the outer overhanging portion of the jaw, down to the root of the tumor, which occupied the whole breadth of the alveolus from one end of the tumor to the other, completely filling the cavity. The base being thus cut off to the level of the socket, I next removed a stump of another tooth, which was now exposed in the root of the tumor, and endeavored with a chisel to shave off the surface of the alveolus, but, as is generally the case, the bone was too hard for this object, and I therefore left it for the application of caustic the next day; and as a vessel of some size came out of the bone at the root of the tumor, and bled a good deal, I placed on it a piece of blue lint, and covered this with a pad of lint, which was kept pressed firmly by the teeth of the lower jaw.

The next day I conveyed some strong nitric acid, by means of a pointed piece of wood with a little linen tied on it, to the base of the tumor within the socket, till it bled too much for more to be applied on that day.

I use the nitric acid, but I do not know that it materially signifies which caustic you employ; but in such a situation as this, within the mouth, you can more easily regulate the acid, as it does not spread beyond where you apply it, and even this effect you can directly stop by a little chalk rubbed on it. Potassa fusa, on the other hand, is very deliquescent, and is carried by the blood about the neighboring parts, so as to injure them, even in spite of vinegar, which you should always have at hand to neutralise the alkali, by means of a little sponge or lint on a forceps or stick. The actual cautery would also do for a superficial part, but not for the hollow of the socket, and I doubt whether the nitric acid would easily have acted on the bottom of this cavity. I should therefore probably have applied a little chloride of zinc on a piece of lint, and forced it in by a compress of lint, just as I checked the hæmorrhage by pressure, putting some soda or lime on the lint, to prevent the deliquescent salt from doing any harm. I found, however, when I next went into the ward, that the patient had gone home, impatient to return to her children, and perhaps rather frightened at the idea of the caustic being repeated, now that she has, as she thinks, got rid of the tumor. It is probable, therefore, that the disease will return, for which she will have herself only to blame, as she has been informed of the chance she has incurred.

